# Quality Assurance of Multiport Image-Guided Minimally Invasive Surgery at the Lateral Skull Base

**DOI:** 10.1155/2014/904803

**Published:** 2014-07-03

**Authors:** Maria Nau-Hermes, Robert Schmitt, Meike Becker, Wissam El-Hakimi, Stefan Hansen, Thomas Klenzner, Jörg Schipper

**Affiliations:** ^1^Chair for Metrology and Quality Management, RWTH Aachen University, Steinbachstr. 19, 52074 Aachen, Germany; ^2^TU Darmstadt, Graphisch-Interaktive Systeme, Fraunhoferstr. 5, 64283 Darmstadt, Germany; ^3^Hals-Nasen-Ohren-Klinik, Universitätsklinikums Düsseldorf, Moorenstr. 5, 40225 Düsseldorf, Germany

## Abstract

For multiport image-guided minimally invasive surgery at the lateral skull base a quality management is necessary to avoid the damage of closely spaced critical neurovascular structures. So far there is no standardized method applicable independently from the surgery. Therefore, we adapt a quality management method, the quality gates (QG), which is well established in, for example, the automotive industry and apply it to multiport image-guided minimally invasive surgery. QG divide a process into different sections. Passing between sections can only be achieved if previously defined requirements are fulfilled which secures the process chain. An interdisciplinary team of otosurgeons, computer scientists, and engineers has worked together to define the quality gates and the corresponding criteria that need to be fulfilled before passing each quality gate. In order to evaluate the defined QG and their criteria, the new surgery method was applied with a first prototype at a human skull cadaver model. We show that the QG method can ensure a safe multiport minimally invasive surgical process at the lateral skull base. Therewith, we present an approach towards the standardization of quality assurance of surgical processes.

## 1. Introduction

True obligatory standards for surgery do not exist. The operation rather depends on other skills like the experience or education of the surgeon or technical and clinical infrastructure. Routine surgical procedures at the lateral skull base depend on stepwise exposure of landmarks due to the individual anatomy of each patient within the temporal bone like the sigmoid sinus, horizontal semicircular canal, or boundary to the dura mater. In this context a large opening cavity is performed by the surgeon to reach a distinct target. The obligatory drilling is time consuming and requires an appropriate skin incision. Other areas of surgery revealed clear benefits of minimally invasive procedures like shorter length of hospital stay, less postoperative pain, earlier postoperative recovery, and a lower complication rate compared to open surgery [1, 2]. In this connection, different procedures at the lateral skull base (e.g., insertion of an electrode during a cochlear implantation or removing a tumor) could be performed without a conventional mastoidectomy or other extensive drilling procedures of the temporal bone. The feasibility of a single-port approach was shown by several groups in preclinical setups [3–8]. Multiport image-guided minimally invasive surgery at the lateral skull base is an innovative increment of existing single-port minimally invasive—or “keyhole”—surgery. The use of multiple ports will enlarge the surgical possibilities when targeting a certain anatomy within the temporal bone. Thus, two ports can be used for instruments like a suction device plus forceps or other microinstruments, while the third port is used for an endoscope to visualize the surgical steps. Due to the high anatomic complexity of the temporal bone and its neighboring structures, a strict risk management is necessary to avoid the damage of critical neurovascular structures during the drilling process. This includes not only the drilling itself but also the entire preoperative and intraoperative management.

For medical products risk management as a part of the quality management system is mandatory as stated, for example, by the US Food and Drug Administration (FDA) [9], in the German Medical Devices Act [10] and by the International Organization for Standardization [11]. Concerning the quality assurance of a surgical process, so far there is no standardized method applicable independently from the surgery. An approach to the evaluation of surgical innovations can be found in a series in the Lancet journal [12–14]. The authors propose the so-called IDEAL paradigm which focusses on establishing new surgical procedures and on showing their efficacy by systematic studies. Still, this proposed procedure does not specifically include presurgical investigations or risk assessments. An approach to the calculation of probability of a successful surgery was proposed by Noble et al. [15]. It takes into account the drill's positioning error at the target. The probability of success is calculated before the surgery and estimates the risk regarding this specific aspect; thus it is not used to secure the whole process chain. Besides, functional and cosmetic results of a surgery are evaluated by the patient and the estimation of a surgery's success is mainly still executed by the surgeon alone. The surgical process is characterized by “standardized” medical access paths, which are defined based on the anatomy and the surgeons' experience. Therefore, in times of increased technology in medicine, an advancing urge to standardize preoperative and intraoperative processes is stated.

As stated above using the example of medical products, in the manufacturing industry in which, for example, product development processes run repeatedly, there exist a variety of methods to manage risks. Possibilities are, for example, to apply methods for fault prevention and to ensure capable processes. One approach to secure process chains is the method of quality gates (QG). QG are defined measuring points that divide the process into different phases or sections. They cannot be surmounted by process outcomes which do not meet previously defined requirements. Interim process results are evaluated regarding the fulfillment of these requirements at each quality gate [16]. This is also the main difference between quality gates and milestones. Milestones are often linked to a specific time and state, for example, that a task has to be finished by a specific date. They primarily define what needs to be done and usually do not indicate who has to provide the information and who is responsible for the process. This last aspect also applies for checklists. The quality gate method aims for measurable criteria and clear responsibilities and has therefore become an integral part of product development and production ramp-up, for example, in the automotive industry, and has also been the object of research for several years [17–19].

In this paper we adapt and apply the quality gate method to multiport image-guided minimally invasive surgery at the lateral skull base in order to secure the innovative surgical process. The defined quality gate requirements are evaluated during a first prototype surgery at a human skull cadaver model.

## 2. Materials and Methods

According to Pfeifer the method of quality gates can be structured into six steps [16].

1. Division of the entire process into natural process sections.
2. Development of measuring points (including the definition of requirements that need to be fulfilled).
3. Development of quality management plans.
4. Monitoring of process progress.
5. Monitoring of measuring points.
6. Synchronization of progress.

In this work the six steps of the QG method have been adapted and applied to a multiport image-guided minimally invasive surgery at the lateral skull base. The team that has elaborated the quality gates consists of otosurgeons, computer scientists who focus on medical imaging and image processing, and engineers from the field of metrology and quality management. In the following, we describe each step and how the QG method has been transferred and adapted accordingly.

**Figure 1:**
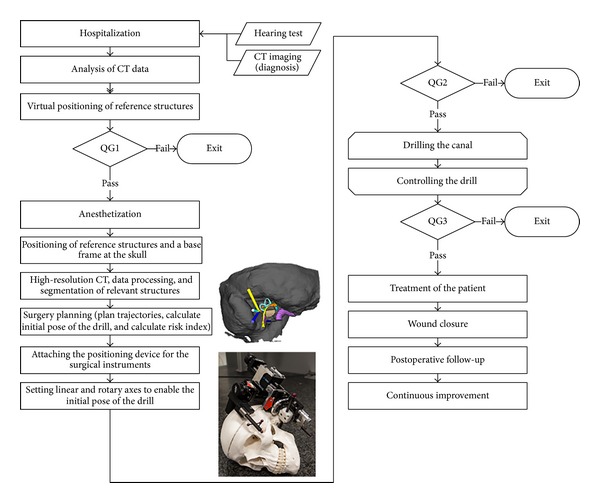
Process for image-guided minimally invasive surgery at the lateral skull base divided into four phases by quality gates. If a quality gate cannot be passed the surgeon should exit the minimally invasive procedure and switch to an established invasive approach.

In the first step of the QG method the process is defined and divided into several sections. If a standard operating procedure (SOP) exists that describes the relevant process, the description of the process and its division into sections can be based on the SOP. For the minimally invasive process that is analyzed here, no SOP exists. Therefore, three QG are defined in an interdisciplinary team which divide the process into four sections (Figure 1). In the second step the requirements that need to be fulfilled at each QG, the QG criteria (Table 1), are defined. This includes that the “customer” and “supplier” for each piece of information are identified. For surgical processes the “customer” of information is the responsible surgeon. The “supplier” is defined for each QG criterion. In order to ensure that everyone accepts the criteria it is important to involve the responsible people in the definition process, for example, in a workshop. For minimally invasive surgery at the lateral skull base the QG criteria was developed in a team of otosurgeons, engineers, and computer scientists. Thus, the person who will carry out the procedure later as well as experts for different technical details are included. The level of detail of the QG criteria is also defined in this group. It should be chosen in such a way that all crucial aspects for the process are covered on the one hand, but on the other hand it should not be too detailed or repeat existing process descriptions to ensure acceptance and applicability to clinical practice.

Up to the first defined QG for this surgical process, the patient has not been exposed to additional radiation compared to the standard procedure. This is the first natural break point of the process. Before anesthetization it is therefore checked if the available data suggests that a minimally invasive surgery could be possible for the given patient. Furthermore, the patient should know about potential risks. These aspects are considered in the defined QG1 criteria. In the section between QG1 and QG2 the surgery is planned in detail which includes a high-resolution CT scan of the temporal bone, the segmentation of relevant structures [20], the planning of trajectories [21] that meet in the target area, and the setting of a positioning system for the drill [22]. Before the actual drilling process starts, QG2 ensures that the risk of damaging sensitive structures is justifiable by calculating the so-called therapeutic risk index (TRI) [22, 23]. This index is based on standards and guidelines from production metrology that have been transferred to the medical domain: the TRI takes into account the distance from the planned trajectory to the next sensitive structure, the uncertainty of CT imaging, and segmentation of the relevant sensitive structure as well as the uncertainty of setting the initial pose of the drill. With the third QG it is checked if the drill canals have been applied according to the planning and if no sensitive structures have been damaged (QG3 criteria).

In the industrial environment people try to avoid that “customer” and “supplier” are the same person in order to ensure a control function. Looking at a surgical process it is obvious to define the responsible surgeon as the process owner and the “customer” of information. As the surgeon is also responsible for supplying information it is recommended to involve a second person in the decision regarding the requirements' fulfillment. This can, for example, be another doctor for the QG1 criteria, a nurse trained for surgical environment for the QG2 criteria, or a resident for the QG3 criteria.

The third step in the quality gate method is the development of a quality management plan. The necessary steps to fulfill QG criteria and pass the gate are described in the process description. When transferring the method to surgical processes it is necessary to define what has to be done if QG cannot be passed. In our current application it is decided that the intervention needs to be switched to a classical, nonminimally invasive approach which we indicate as “exit” (Figure 1).

The fourth step—monitoring of the progress—ensures that deviations from the requirements or parallel activities are recognized and harmonized at an early stage, also between the defined measuring points. This aspect is especially important if several processes run in parallel for a certain time and need to be linked later. Since our current application does not include parallel running processes, this step is neglected in this example.

In step five it is monitored if the target for each process section has been achieved. To do this, the “supplier” gives an evaluation regarding the degree of fulfilment of the defined criteria to the “customer.” If the “customer”—in this example the surgeon—agrees that the aim has been achieved, the process section is concluded. Step six should ensure to document and revisit acquired knowledge so that it can be used to improve future applications.

With the above definitions and descriptions we have established the quality gates to secure the complete process chain in our example.

## 3. Results and Discussion

**Figure 2:**
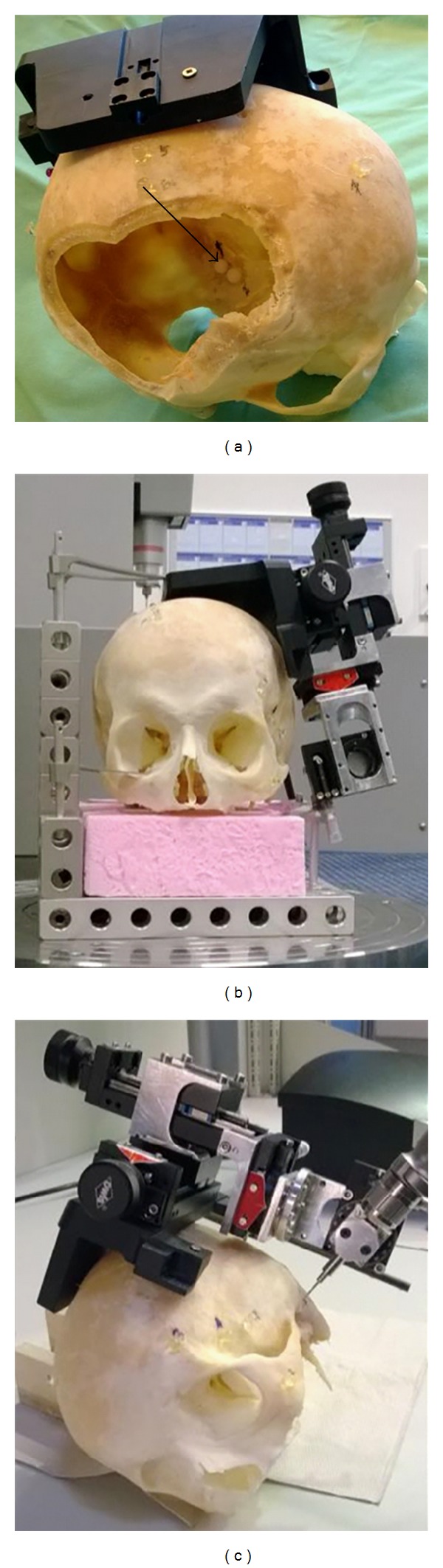
Skull used for prototype evaluation with attached base plate on the left cadaver site, reference structures on the outside of the skull (arrowhead), a target structure inside the scull (arrow), which was placed after opening the right parietal region of the head (a); on the CMM (b), and during the drilling process (c).

In order to evaluate the previously defined QG criteria, this minimally invasive surgical process has been applied for the first time with a human skull (Figure 2). The target point was set at the porus acusticus internus since this is a common surgical target for tumors of the inner ear canal and cerebellopontine angle (neuromas). A second marker was set anteriorly as a reserve but was not used in the described setup. To validate the results experimentally (Figure 1), we performed additional measurements, obviously not possible on real humans: the position of the reference structures and the target area have been measured with a coordinate measuring machine (CMM) giving accurate space coordinates, which are important to ensure traceable measurements. The following changes to the process were made for the experimental validation.

- Glass markers were glued to the skull to ensure a better contrast of reference structures in the cone-beam projections acquired with a C-arm system. They have a slightly higher attenuation factor as bone and therefore can be easily localized in the 2D projections. In addition, they are not producing strong streak-like artifacts, which are typical for metal fiducials. Two additional plastic markers were positioned in the skull, one in the target area and one close to it. These two markers have a relatively low attenuation factor and are therefore ignored during the automatic marker detection process. Nevertheless, they are well recognizable in the reconstructed 3D volume, so that they can be used to define the target point.
- In order to calculate the drill's positioning error, the actual drill position has to be measured and matched to the planned trajectories. Therefore, at least two projections have to be acquired to compute the spatial location of the instrument. The scanner acquisition direction should thereby be perpendicular to the instrument main axis and the angle between the two projection planes should be maximized, with 90° being the ideal value. A 2D/3D registration was performed to align the 2D projections to the preoperative CT data. This was done in two steps: a point based registration followed by an intensity based one. First, the fiducial markers were automatically segmented in the 3D preoperative volume based on knowledge about marker form, size, and attenuation. The challenging task of localizing markers in 2D projections, in presence of form distortion and structure overlapping, was carried out using a recently developed method called particle path segmentation. The achieved fiducial localization error in this step was—mean (std)—0.059 mm (0.062 mm). The point correspondence and the pose estimation were solved with the SoftPosit method [24], which combined the iterative softassign algorithm and the iterative POSIT method. The first algorithm aimed to find correspondences and the second aimed to compute object pose by assuming a full-perspective camera model. A refinement of the estimated projection parameters was then performed by an intensity based registration [25], achieving a target registration error of 0.13 mm (std: 0.02 mm). After segmenting the drill in both projection images, the drill axes were back projected based on the estimated projection parameters. The intersection of the back-projected lines defined the drill position relative to the preoperative data. Lastly, the target localization error was computed as the Euclidean distance between the drill tip and the correspondent planned target.

As a skull was used for this first evaluation of the QG method it occurs that some defined QG criteria cannot be fulfilled because they are not applicable in this setting or because the information is missing. This is, for example, the case with criteria (1.1) and (1.4). For each of these criteria it has to be decided (in the team) whether it can be substituted by other requests (like (1.1), see below) or if it is not applicable and does not need to be considered. If a criterion that cannot be substituted and cannot be neglected is not fulfilled the surgeon should switch to an invasive approach as the process is not secured.

Regarding QG1, all applicable and relevant requirements are fulfilled.

 (1.1) The “patient” does not have a patient chart and was not accompanied with additional information. Usually this would mean a failure to pass QG1. The missing anamneses, clinical examination, hearing test, CT scan, and decision about the necessity of a surgery are in this case substituted by the determination of the target area carried out by an otosurgeon.
 (1.2) The defined target area, the porus acusticus internus, is generally accessible by minimally invasive surgery.
 (1.3, 1.4) These criteria are not applicable for the process with the defined changes as no CT data from previous tests is available and the “patient” cannot be informed.

Therefore, the process can be continued. For this example, the first two QG2 criteria are fulfilled.

 (2.1) The reference structures were fixed to the skull (Figure 2(a)).
 (2.2) High-resolution images have been acquired and are available for further processing.

Criterion (2.3) cannot be fulfilled entirely: the risk structures are segmented, drill trajectories are planned, and anatomical structures as well as reference structures are clearly visible. A TRI of −0.44 is calculated. Based on the definition of the TRI, an index of at least 1 is recommended [23]. This means that when taking into account the previously assessed uncertainties of the initial positioning of the drill and the medical imaging the available distance from the planned trajectory to the next sensitive structure is too small. As this is an index for the capability of the process, an appropriate TRI is essential for the patient's safety. Therefore, the minimally invasive procedure would not be applied in this case if it was a real patient.

In order to be able to evaluate also the QG3 criteria and taking in mind that no real patient is used in this stage, the process was continued, although the risk of damaging the semicircular canals is high. The device for setting the drill to its initial position is fixed to the skull and the axes' parameters are set according to the plan. If these settings are checked and are correct, criterion (2.4) can be fulfilled.

The QG (3.1) criterion is not fulfilled, as the position of the drill was not checked during the drilling process. However, it could be shown that this process step can be successful when computing and matching the drill position to the planned trajectories as mentioned above. Therefore, criterion (3.1) was substituted by this experiment.

The target area, represented by the middle of a plastic ball in the inner auditory canal, was hit with the drill. This means that the inner auditory canal has been reached and QG (3.2) criterion is fulfilled.

Criterion (3.3) is not fulfilled as the semicircular canal was damaged. We expected this due to the previously calculated TRI. Therefore, the decision against a minimally invasive procedure based on the TRI would have been correct in this case and the applied QG method would have succeeded in preventing the damage of a sensitive structure.

## 4. Conclusion

In this paper we show that the quality gate method according to Pfeifer can be transferred to multiport image-guided minimally invasive surgery at the lateral skull base in order to ensure the quality of the surgical process. As this method is established in different fields of industry it is a reasonable possibility to also secure a surgical process chain. Although we focus on one example of multiport surgery at the lateral skull base, this method can also be applied to other single-port surgeries at the head. Necessary changes would be the definition of measuring points and their requirements. In comparison with other methods like checklists [26] or milestones that are also used to standardize processes and to prevent safety hazards, quality gates go one step further by stating responsibilities and by establishing a control function by defining “customers” and “suppliers” of information. Of course, it would be possible to extend checklists in such a way but it is usually not done. The control function is an important aspect of the QG method. Therefore, we ensure this in our example by the requirement of a four-eyes-principle if the “customer” and the “supplier” are the same person.

In an expert team we elaborated the quality gates for multiport surgery at the lateral skull base and applied this method to a cadaver skull for the first time. Furthermore, we showed that the defined measuring points and requirements are useful to ensure a safe minimally invasive surgical process at the lateral skull base. This work is a first approach towards the standardization of minimally invasive surgery which can eliminate or at least reduce potential errors. Therewith, the quality gate method can help to reduce the risk of such a surgery for the patient. For complex surgical processes especially as described in our example, when the surgeon cannot rely on his knowledge and skill alone to carry out the procedure safely but needs software and technical systems for support, the quality gates can help to secure the process when implemented in clinical practice. In order to achieve implementation in clinical practice it is important to integrate the responsible people into the process, for example, during workshops, and to ensure that the defined QG criteria do not contradict existing documents and do not extend the procedure too much.

## Figures and Tables

**Table 1:** Catalogue of criteria for the quality gates of a minimal invasive image-guided surgery at lateral skull base. The customer is the responsible surgeon as he/she is responsible for the outcome of the surgery.

		Supplier
	*QG1 Criteria *	

(1.1)	Presence of all documents of diagnosis: (i) anamnesis, clinical examinations, (ii) indication/necessity of surgery is stated in the patient chart, (iii) hearing test, (iv) CT data set	Patient chart

(1.2)	In principle the target area is accessible by minimally invasive surgery	Surgeons

(1.3)	Available CT data suggests based on the patient's anatomy that it is possible to position drill trajectories. Sensitive structures are not unusually close	Surgeon/radiologist

(1.4)	Patient has been informed of advantages and disadvantages of a minimally invasive surgery. The information is available in the patient chart	Patient chart

	*QG2 Criteria *	

(2.1)	Reference structures have been fixed firmly to the patient's skull	Surgeon and residence

(2.2)	High-resolution CT images have been taken and are available	Radiology

(2.3)	High-resolution images have been processed. Risk structures (including cochlea, semicircular canals, facial nerve, chorda tympani, ossicles, internal carotid artery, and internal and external auditory canal) are segmented and drill trajectories are planned; anatomical structures and reference structures are clearly visible. The therapeutic risk index (TRI) is larger than or equal to 1. The uncertainties of the navigation process and the medical imaging have been taken into account for its calculation	Software

(2.4)	The mechanical positioning device to adjust the drill has been attached to the skull. The axes' position (linear and rotary axis) has been checked based on their scale and the calculated positions from the software	Surgeon and resident

	*QG3 Criteria *	

(3.1)	The continuous process control (using a C-arm) during insertion of the drill has not shown any abnormalities	Surgeon

(3.2)	The target area has been reached. The surgeon can insert an endoscope and has visual contact to the target area. The target area can furthermore be reached by a surgical instrument	Surgeon and resident

(3.3)	No vital structures have been affected (C-arm scan shows no damage of defined sensitive structures and heart rate has been normal)	Surgeon, anesthetist
